# From Mixing methods to the logic(s) of inquiry: taking a fresh look at developing mixed design studies

**DOI:** 10.1080/21642850.2018.1515016

**Published:** 2018-09-06

**Authors:** Rachel L. Shaw, David R. Hiles, Karen West, Carol Holland, Holly Gwyther

**Affiliations:** a Psychology Department, School of Life & Health Sciences, Aston University, Birmingham, UK; b Aston Research Centre for Healthy Ageing (ARCHA), Aston University, Birmingham, UK; c Centre for Counselling & Psychotherapy Education (CCPE), London, UK; d School of Languages & Social Sciences, Aston University, Birmingham, UK; e Centre for Ageing Research (C4AR), University of Lancaster, Lancaster, UK

**Keywords:** Mixed methods research, mixed design research, paradigm, healthy ageing, extra-care housing

## Abstract

**Objective:** This invited paper offers an innovative framework for mixed methods research design.

**Method:** We propose the adoption of the Model of Disciplined Inquiry, a five-component model that focuses on the research question(s) rather than the type(s) of data collected. This pluralist model firmly anchors the research design and paradigm assumptions in the research question(s). Decisions about an appropriate research strategy are made in line with those assumptions. We propose three logics of inquiry to help articulate the processes involved in making sense of findings and their relationship to theory.

**Results:** The Model of Disciplined Inquiry is demonstrated by applying it to the framework to a longitudinal study and describe our decision-making processes at each component stage. The results support the arguement in favour of shifting the focus away from the types of data generated (i.e. qualitative or quantitative) and relatedly a move away from mixed methods research to mixed *design* research.

**Conclusion:** We conclude the paper with some challenges experienced in the example study and some challenges yet to be resolved.

## Introduction

There is now a major interest in the possibilities of using mixed methods research (MMR) in the human and social sciences (Creswell, 2011; Teddlie & Tashakkori, 2011), which has particular relevance to the fields of health psychology and behavioural medicine, where both qualitative and quantitative data can be collected in abundance.

As a research strategy, MMR first developed out of the process of triangulation where two or more research design approaches were used to study a single phenomenon of interest. This strategy places the emphasis on a practical and pluralistic problem-driven approach to research (Denscombe, 2008). Over the past few decades, MMR, has become a major focus of interest within psychology (Yardley & Bishop, 2008, 2017). However, this interest has arisen largely from qualitative researchers in psychology, while being somewhat ignored by quantitative researchers in the same field (Frost & Shaw, 2015; Yardley & Bishop, 2008, 2017).

Historically, MMR in psychology became equated with research designs that utilised both major traditions of inquiry that involved collecting *both* quantitative and qualitative data within a single empirical study (Shaw & Hiles, 2017). The legacy of this is that the kind of data collected has become the focus of attention, which then raises problems regarding how different types of data can be reconciled, or ‘merged’ into an overall conclusion for the research study. The integration of different kinds of data embedded within different and potentially ‘incompatible’ philosophical positions has long been a challenge for MMR, but one that is now being addressed (e.g. Hathcoat & Meixner, 2017).

In this paper, we support recent arguments (e.g. Howes, 2017; Hathcoat & Meixner, 2017) which underline the need for transparency in the philosophical positioning of MMR and the associated philosophical reasoning for the choices of methods used. This special issue provides a brief introduction to MMR detailing some of the philosophical debates around the use of this method. We then offer an innovative framework for MMR, based on the Model of Disciplined Inquiry (Hiles, 2014; Shaw & Hiles, 2017) with a worked example from a recent longitudinal study, which illustrates methodological developments in the fields of health psychology and behavioural medicine.

## An introduction to MMR and its philosophical challenges

Creswell (2008) offers the following definition of MMR, which was adopted by the *Journal of Mixed Methods Research*:

Mixed methods is defined as research in which the inquirer or investigator collects and analyses data, integrates the findings, and draws inferences using both qualitative and quantitative approaches or methods in a single study or program of study.

Such a definition is useful, but works only in the recognition and categorisation of different research methods, and does not acknowledge the controversies that MMR raises. Later, Creswell (2011) discusses eleven controversies ranging from *The changing and expanding definitions of mixed methods research* to *What value is added by mixed methods beyond the value gained through quantitative or qualitative research?* The recurring themes running through these controversies concern what is really driving mixed methods, what precisely is being mixed, and offering perhaps the greatest challenge, whether the different paradigm assumptions underlying the different ‘methods’ of research involved can ever be properly reconciled.

The last of these themes, the issue of paradigm assumptions is probably the most testing challenge to a coherent rationale for MMR (Hathcoat & Meixner, 2017). In his seminal work, *The structure of scientific revolutions* (1962), Kuhn defined a paradigm as a set of recognised scientific assumptions that provide model problems and solutions for a community of researchers. In simple terms, a paradigm is a type of intellectual framework or theory, a ‘worldview’ developed by a research community. Kuhn’s notion of scientific revolutions described the phase of ‘normal science’ where these assumptions are implicit. Over time, new ideas develop which leads to the development of new paradigms, and ultimately the possibility of a ‘paradigm shift’.

Guba and Lincoln (1994) suggested that a chosen *paradigm* rests on certain very basic questions, or assumptions. These concern: the *ontological* (i.e. what is there to know?); the *epistemological* (i.e. what is the nature of knowing?); and the *methodological* (i.e. how do we add to our knowledge?). Later, in Lincoln and Guba (2000), prompted by Heron and Reason (1997), they added the *Axiological* (i.e. what is of value, what it is that is worthwhile to know). These characteristics create our worldview. Based on these beliefs, Guba and Lincoln (1994) distinguish between four major inquiry paradigms for human science research: *positivism, post-positivism, critical theory* and *constructionist*.

Applying these notions to psychology, the *Paradigm Wars* (Oakley, 1999) arguably could chart the shift from psychology as a positivist behavioural science (emphasising empirical evidence) toward the post-positivist and pluralist approaches (where observations may be fallible, phenomena are complex and there are benefits from using different lenses to explore a dataset) we see today in research design, specifically in MMR. Further, the *Paradigm Wars* (Oakley, 1999) refers to the idea that qualitative research rests on a starkly different set of basic underlying assumptions from that of quantitative research. These different approaches result in a range of hurdles that any MMR researcher will need to cross.

The first hurdle is that qualitative and quantitative data are seen as incommensurate, like oil and water they do not mix well. Recent work on the integration of different approaches, i.e. pluralism (e.g. Frost, 2011) is sympathetic to the openness supported by Greene (2007) and Howes (2017), which calls into question the ‘problem’ of incompatibility. Nevertheless, beliefs about associations between the type of data used and the study’s utility persist.

The second hurdle relates to the methods of reasoning used by each approach. The quantitative approach to research has become closely associated with the traditional scientific method of hypothesis testing, which is essentially deductive and confirmatory in its approach. By contrast, the qualitative approach generally employs an inductive and exploratory approach. This inductive approach starts with particular (local) instances/events, data are collected in response to a carefully formulated research question, the data are then analysed for patterns and structures of meaning, out of which theoretical constructs can emerge.

A third hurdle involves how quantitative research is basically regarded as *theory-driven*, with theoretical or model-based predictions generating hypotheses to be tested by perhaps an experiment or a field study, with the outcome that the hypothesis is either accepted or rejected, and the theory/model is supported, or undermined, potentially leading to new or adjusted theories or models.

In contrast, qualitative research is more associated with ‘discovery’ science in new and innovative fields of study (Hiles, 2014) and is regarded as primarily a *data-driven* approach offering scientists ways to study complex, emergent/chaotic phenomena that may be inherently unpredictable (Hiles, 2016), which is inevitably the case with much human behaviour and experience. The outcome of this data-driven approach is the emergence of new insights, new theoretical constructs, and new theory. This is also especially useful when new fields of study are being developed, where methods of exploration and discovery offer ways of adding to human knowledge. For example, research in the human sciences is not just about ‘observable’ phenomena, the ‘voice’ of the participant in the context of their experience can serve to contextualise other observations.

However, these assumptions are somewhat simplistic and do not recognise how methods are used in practice. In particular, health psychology and other applied health-related research have embraced MMR and have adapted well to different ways of conducting qualitative analysis, which may involve the ‘traditional’ inductive, data-driven approach or a more deductive, theory-driven approach (e.g. Braun & Clarke, 2006; Terry, Hayfield, Clarke, & Braun, 2017). Furthermore, framework analysis (Gale, Heath, Cameron, Rashid, & Redwood, 2013) often employed in health services research, offers the opportunity to incorporate a theoretically informed *a priori* framework in an analysis involving both induction and deduction. One example is the Theoretical Domains Framework (TDF: Cane, O’Connor, & Michie, 2012) of which an applied example can be found in Shaw, Holland, Pattison, and Cooke (2016). Thus, mixed methods offer a striking opportunity to combine the strengths of these two distinct approaches to scientific research within a single study.

## The model of disciplined inquiry

The model of Disciplined Inquiry (Hiles, 1999, 2006, 2014; Shaw & Hiles, 2017) offers a five stage, comprehensive and generalised approach to MMR that emphasises the research *designs* used in a research study, that stresses the underlying logic(s) of inquiry.

The model is fundamentally pluralistic in approach. Polkinghorne (1983) was an early advocate of a pluralist approach, specifically at the level of the paradigm.

The difficulty for human science arises, not from a need to change from one paradigm to another, but from a need to resist settling down to any single paradigm. (Polkinghorne, 1983)

Polkinghorne argues that it is the *paradigm(s)*, rather than the methods, that are crucial to the research process, and we propose that this is critical in re-thinking MMR and placing the focus squarely back on the research question. Certainly, this model avoids current preoccupations with ‘method’, particularly the categorisation of MMR designs (e.g. exploratory, explanatory, sequential, embedded etc.,) and forms of integration (e.g. mixing, merging, connecting, and embedding) at different stages of data collection, analysis and interpretation (cf. Creswell, 2008), rather the model focuses on the specifics of the research objective and nature of the research question. However, the model does incorporate both major *traditions* of inquiry (i.e. qualitative and quantitative methods).

The term, *Disciplined Inquiry*, adopted in naming this model, is not new. Educational researchers established the term and it was introduced into the psychology literature by Braud and Anderson (1998). It is a useful term to characterise the general features of an inclusive, structured framework for scientific research and enables meaningful debate about the nature of MMR design.

**Figure 1.**
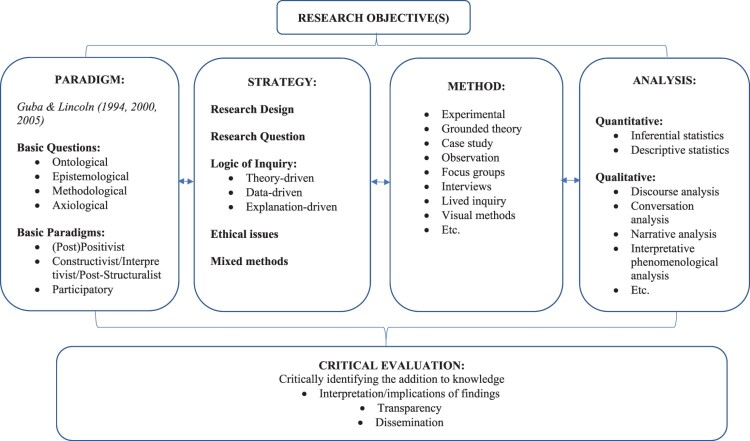
The model of disciplined inquiry.

The model of *Disciplined Inquiry* takes the research question as its starting point. While we appreciate that the MMR tradition has also had the research question at its heart in establishing which methods are appropriate, we argue that this needs to be made more explicit in the development of a research design. The Model of Disciplined Inquiry treats the inquiry process as one that is open to a variety of *assumptions, choices, procedures, data analysis techniques*, and *critical reflections.* These are characterised formally as five components: *Paradigm*, *Strategy*, *Method*, *Analysi*s and *Critical Evaluation*, illustrated in Figure 1. Thus, the basic *Paradigms* that research can follow, lead to a range of different *Strategies* of research, drawing upon a rather large range of different *Methods* for collecting data, a range of approaches to the *Analysis* of data, and, finally, consideration in the *Critical Evaluation* and dissemination of the research findings. In summary, the model of Disciplined Inquiry, developed out of the work of Guba & Lincoln, has these basic features:

- a five stage framework, with clear research question(s) in mind, that embody a logical progression from making assumptions through to critical evaluation, that are overlapping and mutually interdependent;
- provision for three basic paradigms of inquiry that are to be used pluralistically, with an explicit provision for mixed designs and methods;
- a set of core principles for all human inquiry that constitutes a fundamental logic of inquiry.

## The ARCHA and extracare collaborative study

**Figure 2.**
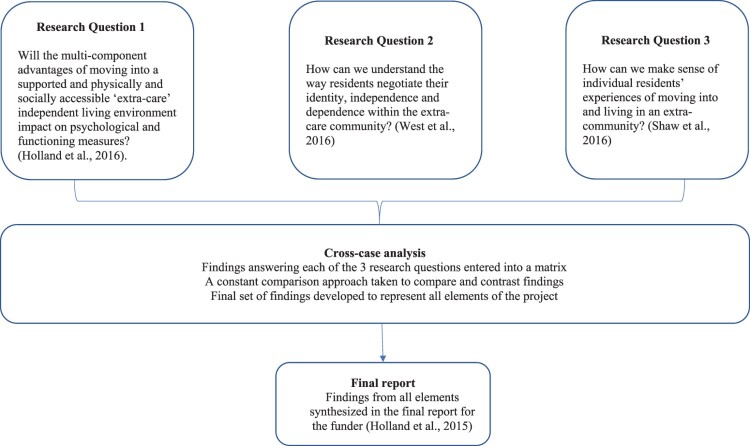
The ARCHA-ExtraCare Project as a convergent parallel design.

This article describes how the model of Disciplined Inquiry was applied to a longitudinal MMR project that adopted a convergent parallel design (see Figure 2). The MMR project was a collaboration between Aston Research Centre for Healthy Ageing (ARCHA) and ExtraCare Charitable Trust. Details of the study can be found in Box 1. The project examined the notion of healthy ageing through the psychological and functional impact of an ‘extra care’ independent living environment. Detailed results are reported elsewhere (Holland et al., 2015; Holland et al., 2016; Shaw, West, Hagger, & Holland, 2016; West, Shaw, Hagger, & Holland, 2016). This article has a purely methodological focus and provides an overview of each of the five components of the model of Disciplined Inquiry, alongside the rationale for each component selection by the research team.

Box 1. **The ExtraCare Case Study – research setting, objectives and design.**

**Research Setting:** ExtraCare is a charitable organisation that builds and operates retirement villages for older adults. It aims to provide the ‘housing, care and support needs of older people while helping them maintain independence in their own private accommodation’ (Netten, Darton, Baumker, & Callaghan, 2011, p. 4). ExtraCare offers an accessible environment, opportunities for social interaction, a varied provision of physical, social and intellectual activities, while also providing ‘extra-care’, whereby a privately funded personalised care service is available for residents, should they require or request it. Well-being advisors (nurses) are also available on request to offer support, for example, in managing chronic illnesses.

**Research objectives:** The objective of the research was to evaluate, measure and understand experiences of extra-care accommodation to determine whether it produces positive outcomes for healthy ageing (Holland et al., 2015p. 14).

**Research design:** A mixed method, convergent parallel design was chosen for this evaluative project. We noted that positive outcomes for older adults could be identified in multiple ways, i.e. in relation to social, cognitive, economic, and health outcomes. We were required to obtain measurable evidence for positive outcomes. We were also required to provide explanations for any positive outcomes identified, i.e. what is the nature of older adults’ experiences in extra-care accommodation. To achieve this, we broke down the study into several parallel research questions.

**Publication Strategy:** A challenge for researchers using multiple methods is publishing the work in a way that values each element and which supports claims made with fully integrated findings. We were able to converge findings into an integrated report, which is available online (Holland et al., 2015; http://www.aston.ac.uk/lhs/research/centres-facilities/archa/extracare-project/). Unfortunately, in order to fulfil our objectives and publish findings in peer reviewed journals we had to split the project into its component parts. This sacrifice was necessary to enable publication and impact.

### The components of the model

#### 1. Paradigm

The First component to be considered in the Disciplined Inquiry Model is the paradigm. Guba and Lincoln (1994) propose that a paradigm of inquiry is the set of basic assumptions that every scientist must make in designing their research. These assumptions are based on their ‘worldview’ and the way in which they have chosen to address the ontological, epistemological and methodological issues. It follows that there is no single paradigm that is the right one, or the wrong one, and many different paradigms can exist alongside each other, and indeed can be combined in a single study, as is the case with the ARCHA collaborative study.

Guba and Lincoln (1994) originally considered four major inquiry paradigms. Within the Disciplined Inquiry model, these four paradigms have been simplified under two broad headings*, (Post) Positivist* and *Constructivist/Interpretivist/Post-structuralist.* Briefly, the *Positivist* paradigm is characterised by a naïve realism, there to be studied and verified by manipulation and experiment (i.e. that scientific experiments can reveal an objective reality or ‘truth’). The *Post-positivist* paradigm rejects positivism and is characterised by a critical realism, whereby the phenomenon of interest is studied by a process of conjecture and refutation (cf. Bhaskar, 1989; Popper, 1989). The *Constructivist/Interpretivist/Post-structuralist* paradigm is characterised by a historical realism shaped by social, political, cultural, economic, ethnic and gender values, where the researcher is seen as a *bricoleur* (literally a ‘jack-of-all-trades’, i.e. piecing together techniques according to what is appropriate at the time) using a range of interpretive theory and practices to make the world visible and understandable (Denzin & Lincoln, 2011, 2018).

The Disciplined Inquiry model adds one further paradigm – the *Participatory* (cf. Heron & Reason, 1997), which despite some overlap does not easily fit with the others. This paradigm provides a proper place for methods of human inquiry such as phenomenological inquiry, heuristic inquiry, auto-ethnography, action research, transpersonal inquiry, and some approaches to narrative inquiry. However, it needs to be noted that the difference between Heron & Reason’s idea of a *participatory inquiry paradigm* and the term *Participatory* used here, is that the emphasis is less on the methodological issues, and more on the fundamentally different kinds of knowledge that are involved concerning the *Ontological* and *Epistemological* issues.

The relevance to MMR is that while different paradigms exist, they do not have to be viewed as rivals, but can be combined within a single research study (as has been argued elsewhere, for example: Johnson & Onwuegbuzie, 2007; Greene, 2007). Expressed quite simply, in MMR it is not that different methods are being mixed, but it is *paradigms*, i.e. ontologies and epistemologies, that are being mixed (for further discussions of pluralistic use of paradigms in psychological research see: Frost, 2011; Frost & Nolas, 2011).

Thus, the first stage of the application of the Model of Disciplined Inquiry to our research project was to determine which paradigms were of relevance with respect to which research question. The details can be found in Box 2.

Box 2. **Component 1 – Paradigm.**

**Research Question 1: Will the multi-component advantages of moving into a supported and physically and socially accessible ‘extra-care’ independent living environment impact on psychological and functioning measures? (** **Holland et al., 2016** **).**

Embedded within this research question is the assumption or hypothesis that living in extra-care accommodation will produce positive outcomes. As such, our paradigm assumptions fitted within a post-positivist framework.

**Research Question 2: How can we understand the way residents negotiate their identity, independence and dependence within the extra-care community? (** **West et al., 2016)**

The second question focussed on the quality of residents’ experiences of moving into and living in extra-care accommodation. We wanted to explore the nature of extra-care accommodation through the eyes of both new and existing residents, to determine how residents negotiated their new living space and how the move might impact on their sense of self. This required an ontological approach sensitive to the complex ways in which a social community develops, interacts and lives. Our assumption was that such a community is actively created by its individual members within the constraints of the physical space of the village, the historical ethos of ExtraCare as an organisation, and the kinds of relationships developed between residents and staff. These assumptions fitted within a constructivist/post-structuralist paradigm because of their focus on subjectivity, discourse and systemic features of ExtraCare. As a team, we were committed to critical realism, i.e. we believed that multiple versions of events co-exist, which means that understanding human experience requires an examination of meanings.

**Research Question 3: How can we make sense of individual residents’ experiences of moving into and living in an extra-community? (** **Shaw, West, et al., 2016**)

The third question required us to focus on individuals’ subjective experiences, how people made sense of their experiences, what about them was shared and what was unique. This question suggested idiography, i.e. making sense of the phenomenon at the individual, case-study level. This was commensurate with a constructivist/interpretivist paradigm.

#### 2. Strategy

The Second component of the Model of Disciplined Inquiry relates to strategy. Strategies of inquiry are concerned with the formulation of the research question(s) and decisions involved in planning the research design, including decisions about the *logic of inquiry*. Strategies provide a crucial bridge between the paradigm(s) on the one hand and the method(s) of data collection and data analysis on the other.

When considering strategies, the formulation of the research question is key. Choices need to be made between three approaches – a *theory-driven* approach (e.g. *hypothesis-testing*, most likely using quantitative methods), a *data-driven* approach (e.g. *grounded theory,* most likely using qualitative methods), and *explanation-driven* approach (e.g. a *case study* is a good example). Moreover, each of these approaches has its own *logic of inquiry*.

The first logic of inquiry (Logic 1) is theory driven. It is driven by *deductive inference*, and is concerned with testing the *prediction*, from theory towards the data (findings) expected and is typical of a hypothethico-deductive study. The second logic of inquiry is data driven. *Logic 2* is driven by *inductive inference*, and is concerned with *generating* from data the new theoretical constructs, with a typical example being a grounded theory study. The third logic of inquiry focuses upon an examination of how well existing theory fits with the data in some way. We call this *explanation-driven*. *Logic 3* is driven by *abductive inference*, and is concerned with the two-way *explanatory relationship* between theory and data. The best example of a Logic 3 design is the case study, where data is collected, not to test an existing theory, or to allow a new theory to emerge, but to explore how existing theories can explain the specific phenomena of the case (where indeed even rival theories can contribute to a deeper understanding of the case). This third type of logic of inquiry is crucial to mixed methodology studies in the field of health psychology and behavioural medicine and frequently arises in research relating to professional practice (e.g. clinical, health, education), in action research, ethnography, and in single-case studies, etc., where the emphasis is on trying to *understand* and *explain*.

It is a central tenet of the Disciplined Inquiry approach that the research question is crucial in identifying the logic of inquiry, as well as identifying the paradigm(s) in planning the research design. Furthermore, where two or more research questions are being combined in a single study, as is the case with an MMR study, it is critical to recognise that it is not specifically methods or data that are being mixed, but it is the *logics of inquiry* that are being mixed. This concept was previously recognised by Onwuegbuzie and Leech (2006) and Robson (2011), but the approach suggested here offers a logical five-step framework, which guides researchers through the design process and helps to place the focus back on research *designs* and logics of inquiry rather than research *methods*.

**Figure 3.**
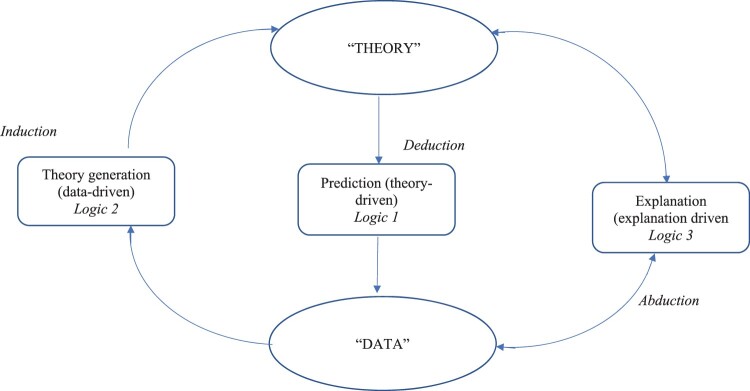
The relation between theory, data and logic of inquiry.

The nature of this underlying three-fold relationship between theory and data rests upon the three basic types of human reasoning: *deduction*, *induction* and *abduction* each corresponding to one of the three distinct logics of inquiry. The relationships between theory and data is illustrated in Figure 3, where the arrows represent the direction of this relationship and correspond to a distinct logic of inquiry.

The notions of *deduction* and *induction* may be very familiar, but *abduction* is not widely understood, and, with a few exceptions, is almost completely overlooked in the methodological literature. *Abduction* is a term which was first introduced by the American philosopher, Charles Peirce (1903), and more recently has been aptly described as: *inference to the best explanation* (Harman, 1965; Lipton, 1991). Peirce distinguished between deduction, induction and abduction in human reasoning, and this has crucial relevance to understanding both everyday human rationality (Hiles, 2009), as well as scientific methodology.

Thus, the Model of Disciplined Inquiry supports a move away from the limitations imposed on research design by the terms ‘qualitative methods’ and ‘quantitative methods’ and towards a terminology that liberates researchers and opens up new possibilities by emphasising *theory-driven, data-driven,* and *explanation-driven* designs*.* Priority needs to be given to understanding the *logic of inquiry* in research, by reducing the focus on the types of data being collected, and instead placing the focus onto the nature of the *research question*, and types of *design* being used. Placing the focus upon *mixed design*, rather than mixed methods, challenges the current arguments for a burgeoning classification of ‘mixed methods’ (cf. Creswell & Plano Clark, 2011), or the idea that only *eight* types of mixed methods are possible (cf. Morse & Niehaus, 2009, p. 25). Such typologies are only concerned with methods of data collection, rather than, as being argued here, the crucial issues relating to the logic of inquiry. The application and rationale of the chosen strategy and logics of inquiry in our example project can be seen in Box 3.

Box 3. **Component 2 – Strategy.**

**Research Question 1: Will the multi-component advantages of moving into a supported and physically and socially accessible ‘extra-care’ independent living environment impact on psychological and functioning measures? (** **Holland et al., 2016** **)**

The chosen paradigm demands the ‘traditional’ theory-driven logic of inquiry (Logic 1), involving deduction to test a hypothesis.

**Research Question 2: How can we understand the way residents negotiate their identity, independence and dependence within the extra-care community? (** **West et al., 2016** **) & Research Question 3: How can we make sense of individual residents’ experiences of moving into and living in an extra-community? (Shaw, West, et al., 2016)**

These research questions both begin with a familiar data-driven logic of inquiry (Logic 2). However, there was an intention from the outset to take an abductive/retroductive approach to the data, i.e. to consider the data generated alongside existing theoretical concepts to help explain them further and to better understand the phenomena of identity, dependence and independence within the context of living in extra-care accommodation. As such, we also moved into an explanation-driven logic of inquiry (Logic 3).

Strategies of inquiry also involve choices that need to be made with respect to acknowledging and dealing with potential bias, with sampling both participants and the phenomena under study, and with choices in respect to how the phenomena are to be studied. Another matter that cannot be overlooked in human research is the range of ethical issues that will need to be raised. Whilst the highest standards of concern with respect to the *potential for harm, confidentiality, informed consent,* and *the application or impact of the research* are required, it is necessary to recognise that these can receive significantly different treatment within different paradigms and strategies of inquiry. For example, specific considerations, especially with respect to confidentiality, must be given to the treatment of data that involves reports of individual experience, single-case studies, transcripts of interview material, etc. Ethical principles developed in areas that have been largely concerned with quantitative analysis, need to be completely re-thought when a qualitative approach or mixed approach to data collection and analysis is being considered because of the different ways in which data are gathered and handled.

#### 3. Method

The Third component in the model is method. Unfortunately, the term ‘method’ has become a source of confusion in the research literature, and especially in qualitative and quantitative texts. It has been used interchangeably when referring to methods of data collection as well as methods of data analysis, and this leads to confusion within MMR.

By contrast, in the Disciplined Inquiry model, the term ‘method’ is used strictly to refer to the ‘method(s)’ for the collection of data, and not to ‘method(s)’ for the analysis of data, or to the type of design being used in a study. Thus, it follows that, by limiting the definition in this way, MMR is reduced to a mere description, and we argue that the term *mixed design research* (MDR), becomes more useful and should replace the term MMR (cf. Hiles, 2014).

Alongside the traditional methods of quantitative research (e.g. experimental, quasi-experimental designs, etc.) with their inherent concerns with controlling variables, random assignment of participants, etc., there is an increasing range of qualitative ways of collecting data, such as interviewing, focus groups, single-case study, lived inquiry, etc. A further matter of concern involves researcher as a participant in the inquiry process, e.g. interviewing, focus groups, action research, participant observation, heuristic inquiry, auto-ethnography, etc. The point is that considering methods of data collection simply in quantitative vs. qualitative terms is limiting. It is quite possible to obtain qualitative data under experimental conditions, and perfectly feasible to obtain quantitative data from a range of interviews. The only constraints are those that follow from assumptions made by the paradigm of inquiry and the choices of research design.

The rationale for our chosen method in the longitudinal study can be seen in Box 4.

Box 4. **Component 3 – Methods.**

**Research Question 1: Will the multi-component advantages of moving into a supported and physically and socially accessible ‘extra-care’ independent living environment impact on psychological and functioning measures? (** **Holland et al., 2016** **)**

As a prediction was made based on previous literature, it made sense to use experimental methods, in this case standardised validated measures of potential positive outcomes. In brief, we chose measures that assessed cognitive function, autobiographical memory, anxiety and depression, instrumental activities of daily living, functional limitations profile, and self-perceived health (see Holland et al., 2016). To determine whether there were changes (and benefits) over time, we needed to take these measures at multiple time points over the 18-month study period and compare them with a (community) control group.

**Research Question 2: How can we understand the way residents negotiate their identity, independence and dependence within the extra-care community? (** **West et al., 2016** **)**

This question focused on the community aspects of extra-care accommodation and so we determined that it was important to observe the interactions between residents. To do this we elected for focus groups with residents who had lived in an extra-care community for some time and some residents who had recently made the move. We also decided to observe the community in action by spending time in the communal areas and with a few residents in their own apartments.

**Research Question 3: How can we make sense of individual residents’ experiences of moving into and living in an extra-community? (Shaw, West, et al., 2016)**

This research question required accounts from individuals about their own unique experiences and their meaning-making processes. To achieve this, we conducted individual semi-structured interviews with residents soon after they had moved in and then twice more over a period of 18 months to get a sense of how their move to an extra-care community developed over time. This longitudinal approach to data generation in a health psychology study about individuals’ experiences of a phenomenon over time can really help with an approach focussed on making sense of how participants make sense of and come to terms with a new living situation, e.g. diagnosis of a new health condition.

#### 4. Analysis

The Fourth component within the model of Disciplined Inquiry is the analytical strategy. By adopting an MDR approach there will be the need to use both quantitative and qualitative approaches to data analysis as appropriate and consistent with the original paradigm(s) of inquiry, with the research questions, research design and the methods of data collection. Quantitative analysis is of course a proven approach with enormous range and application, and will be consistent with a *theory-driven* approach, that draws upon the logic of hypothesis testing.

Qualitative analysis is often consistent with a *data-driven* approach, with data consisting of meanings, accounts and descriptions, open to the process of interpretation, which might involve discourse analysis, narrative analysis, phenomenological analysis, hermeneutics, case study, etc. However, it needs to be acknowledged that the nature of interpretative techniques must inevitably involve the subjectivity and biases of the researcher/team involved in the data analysis. In some areas of research, it may be possible to go a long way in eliminating bias. While in other areas, biases need to be worked with rather than treated as something to be avoided at all cost.

The rationale for our choice of methods in the project can be found in Box 5.

Box 5. **Component 4 – Analysis.**

**Research Question 1: Will the multi-component advantages of moving into a supported and physically and socially accessible ‘extra-care’ independent living environment impact on psychological and functioning measures? (** **Holland et al., 2016** **)**

Methods for this research question generated numerical data that demanded statistical analysis. Each standardised measure had its own scoring guide. To test if there was a difference between the extra-care and control groups, a series of analytic tests were conducted including analysis of variance and covariance, regression modelling and TOBIT analysis^1^ to account for floor and ceiling effects (see Holland et al., 2016).

**Research Question 2: How can we understand the way residents negotiate their identity, independence and dependence within the extra-care community? (** **West et al., 2016** **)**

The methods used generated textual data from focus groups (transcriptions of audio-recordings) and observations (field notes). We began data analysis thematically drawing on inductive reasoning (Logic 2), i.e. prioritising the data and allowing that to direct our meaning-making as analysts. This analysis indicated the significance of how older adults talked about their experiences of living in an extra-care community. Phenomena including independence, dependence, and autonomy were significant. Furthermore, the older adults who took part in this part of the study seemed to enjoy complaining about their life in ExtraCare; they very clearly reported contentment in their extra-care community, but at the same time, they loved to moan about it! We critically reviewed the social gerontology literature to identify a theoretical framework that would help us make sense of these phenomena (Logic 3). The third age/fourth age dialectic (e.g. Higgs & Gilleard, 2015) gave our analysis ‘a certain conceptual anchoring’ (West et al., 2016 p. 7) to help explain what we were finding, following Peirce’s principle of abductive inference. As sociological and psychological scientists, we were invested in post-structuralist discourse theory as a method for making meaning from the ways in which older adults played out this dialectic in their talk about living in an extra-care community (see West et al., 2016).

**Research Question 3: How can we make sense of individual residents’ experiences of moving into and living in an extra-community? (Shaw, West, et al., 2016)**

The methods used in the final research question generated a series of individual interview transcripts for a small case study of residents. Initially inductive inference was used to generate data-driven conceptualisations of the phenomenon. We took an idiographic approach to making sense of individuals’ lived experiences over an 18 month period and so Interpretative Phenomenological Analysis was deemed appropriate (IPA; Smith, Flowers, & Larkin, 2009). The data focused on wellbeing. Themes were developed inductively (i.e. Logic 2) for each individual and compared across the sample (see Shaw, West, et al., 2016). We then, using an abductive strategy, searched for theories that would help explain residents’ experiences of living in extra-care accommodation (Logic 3). To complement the phenomenological method and enhance coherence between the techniques and ontological assumptions, we identified a phenomenological theory, Galvin and Todres (2013) phenomenological theory of wellbeing, which draws on Heidegger’s notion of homecoming to explain the experience of authentic wellbeing. An authentic sense of wellbeing can be experienced once an individual has undergone existential vulnerabilities, such as moving into extra-care because of an acute event, e.g. a stroke. Thus, we employed Galvin and Todres (2013) conceptualisation of wellbeing to engage in a ‘thinking dialogue’ between data generated and phenomenological concepts of wellbeing (full details are in Shaw, West, et al., 2016).

#### 5. Critical Evaluation

The final component of any piece of research must be a critical evaluation of all aspects involved in the inquiry. The guiding principle should be a close examination of how the research question(s) are being ‘answered’, and a critique of the claim that a *contribution to knowledge* has been achieved. This will involve, in principle, three areas of reflection and discussion:

- reflection on the interpretation and implications of the findings;
- establishing the transparency of the assumptions, choices and procedures used in the study, leading to reflection on the strengths and weaknesses of the research design, methods of data collection, analytical approach, the strategic choices made, the possible sources of bias and reliability in the data, and the consequent limitations on the conclusions that can be made;
- reflections involved in presenting the relevance and impact of the research findings (i.e. the axiological values) to others in the wider community, who will in turn bring their own critical powers of reflection to bear on the inquiry.

In writing up an MDR study, there is an obvious requirement to review the existing literature from the widest variety of sources. The basic principle for such a literature review is the need to show how the research findings constitute an addition to knowledge, and how a significant contribution to the topic area is being made. However, in the case of social/human research, using merely the established model for writing-up an *experimental study* is not appropriate.

The problem for an MDR study is that with the *theory-driven* part of the study, a review of the literature is necessary to make a prediction and formulate the hypothesis. While with the *data-driven* part of the study, which is inherently exploratory, and where the logic of inquiry is different, the role of the literature review is to set out the *rationale* of the study, since the study’s findings cannot be predicted, but instead, the theory (or theoretical constructs) emerge from the data. And for the *explanation-driven* part of the study, only after the case material has been collected and summarised, can the constructs and theories be identified that offer some understanding of the case(s) being studied.

Critical evaluation will necessarily raise concerns about the type(s) of data that have been collected. In the case of quantitative data, this invariably raises issues of *validity*, *reliability* and *objectivity*, but in the case of qualitative data a different set of considerations will come into play. Criteria that need to be addressed with qualitative data include: *credibility, transferability, trustworthiness* and *confirmability*. Of course, these issues are not new and various proposals have already been made regarding how best to proceed with quality appraisal of MDR (e.g. O’Cathain, Murphy, & Nicholl, 2008; Collins, Onwuegbuzie, Johnson, & Frels, 2013; Souto et al., 2015). Fundamentally though, in writing up an MDR study, what is required is *transparency* in the various methods of data collection and data analysis, i.e. it is essential in writing-up the research findings that all procedures are sufficiently clear to be followed by someone else. One of the final challenges for MDR studies is in reporting them in an integrated form. Currently, the publishing world prioritises short papers, and journals sometimes prize one approach over another, which inevitably reduces the potential for publishing MDR studies in one place (as was the case for the ARCHA-ExtraCare project).

The key aspects in the critical evaluation of our collaborative research project can be seen in Box 6.

Box 6. **Component 5 – Critical evaluation.**

Transparency and validity were key in this collaborative project between ARCHA and ExtraCare Charitable Trust. Validity was ensured by regular communication and collaboration between the parties, with a steering committee, comprising key members of both teams, tasked to consider interpretations, reflect on any challenges encountered, monitor progress and oversee findings. Both parties worked independently while in partnership to ensure the quality of data generated. Further, each research-active team member kept a reflexive diary, which were used to inspire discussions around emerging themes and to identify applicable theoretical constructs. Reflexivity is a key indicator of validity in research using interpretative methods of analysis, which actively involve the researchers as participants (e.g. Shaw, 2010). After the report was finalised, an action-learning group was formed to ensure that recommendations were implemented at ExtraCare villages going forward (see Holland et al., 2015). Findings were disseminated to stakeholders at specialist conferences and within ExtraCare Charitable Trust, the full report was published online (Holland et al., 2015) and distinct elements were published in peer reviewed journals (see: Holland et al., 2016; West et al., 2016; Shaw, West, et al., 2016).

Answering the multiple research questions in this convergent parallel design produced a set of findings that has had far more utility in terms of informing the introduction of new policies and procedures at ExtraCare and the development of new evidence-based interventions to test in further research than any separate element of the study would have produced alone.

### Ethics statement

The work presented as the example was approved by the University Ethics Committee.

## Discussion

This paper has presented a logical framework for developing *mixed design research* (MDR) studies through the Model of Disciplined Inquiry, aided by a worked example of published research. Central to our proposal is the need to take a fresh look at ‘mixed methods research’ by re-thinking the relationship between the research question, the logic(s) of inquiry, and paradigm assumptions, rather than focusing the debate around the methods used for data collection and the type of data generated. We argue for a change in terminology to ‘mixed design research’ to place the focus squarely back on the paradigm and the logic of inquiry. This is not to say that MMR does not do this already, but we argue that this shift in terminology will make the focus on the research question(s) more explicit. It should be clear that an MDR study is defined as a research programme that involves two or more research questions that involve two or more logics of inquiry. Moreover, we argue for a move away from the labels of qualitative and quantitative research because, again, they simply identify the nature of the data rather than the logic of inquiry employed.

Developing MDR will present challenges to many researchers because it requires additional or different processes and new ways of thinking. We will now outline some of the issues identified in writing the present paper and in the development and conduct of the worked example of the ARCHA-ExtraCare Project.

The Model of Disciplined Inquiry requires researchers to prioritise the research question when starting to think about their design. Many a time students come to us saying, ‘I want to do a qualitative project’ or ‘I want to run an experiment’ before they have even contemplated the detail of their research question. More advanced students and those working in interdisciplinary teams on externally funded projects face similar challenges when working with people who have preconceived ideas about which methods are ‘the best’. For example, the hierarchy of medical evidence prioritises randomised controlled trials of interventions over cohort studies, case studies, and excludes qualitative studies altogether (e.g. Guyatt et al., 1995). Over time, this has created a focus on effectiveness and cost-effectiveness of interventions within healthcare research which prizes a particular strategy, i.e. experimental designs and the logic of deduction. However, this precludes the study of the context in which an intervention is tested, its feasibility and how acceptable service users may find it (Shaw, Larkin, & Flowers, 2014). This narrow definition of evidence is being challenged by the very presence of the debate around mixing methods and the growth of mixed design research (O’Cathain, Murphy, & Nicholl, 2010). Nevertheless, promoting the use of Disciplined Inquiry needs to include education that is sensitive to the history of health psychology and behavioural medicine and which will demonstrate its utility in terms of the added value and impact of MDR.

The first challenge we identified in recommending a Disciplined Inquiry approach to MDR is that it requires some contemplation about the paradigm assumptions imbued within the research questions and the implications of those for choosing an appropriate strategy and logic(s) of inquiry. This deeper thinking about research from the outset will be new to many and can be difficult when working with stakeholders or service users who are always, understandably, very eager to make things happen and not always so enamoured with the idea of theory.

A related challenge is working in a mixed skilled team; quite often in MDR there are experts in specific methods with research assistants working on different elements of the project. This was the case in our study. Although communication channels were open between teams working on the three research questions, there was unsurprisingly more collaboration in the work undertaken to answer the second and third questions due to significant overlap in methodological expertise. The intricacies of the statistical analysis in the first research question were unfamiliar to those working on the latter two and vice versa. Notwithstanding these challenges to our understanding, we were able, as a team, to benefit from the range of expertise we brought to the problem and therefore answer the research questions in ways that added depth of meaning to a large scale, longitudinal programme of research assessments.

The findings from each research question were converged through the development of a matrix and an analysis modelled on meta-synthesis (e.g. D’Avanzo et al., 2017) to identify areas of convergence and divergence across research questions (see Figure 2). We were lucky in the ARCHA-ExtraCare project that the findings from all parts of the project were complementary (details of how the findings across all research questions supported each other are discussed in the full report, see: Holland et al., 2015). The possibility of contrasting findings in MDR could be perceived as a problem. If the MDR involves exploratory and experimental designs the most dominant will usually prevail; in the case of behavioural medicine and health psychology, this is likely to be the experimental element. This could mean that findings from the exploratory work are rejected because they are judged differently in terms of validity, to large data sets of experimental data which are ‘powered’ to demonstrate an effect ‘with confidence’. However, findings from exploratory, smaller scale work may shine a light on nuances of experience which help us understand the context of the experiment, or which perhaps help us devise new research questions to further interrogate the phenomenon instead of taking the results from the experimental work at face value, which may have limitations of different kinds. We argue, like Pluye and Nha Hong (2014), that using both stories and numbers will result in more comprehensive findings.

The weight given to different elements of MDR can be contentious when working within a politicised setting, such as in the ARCHA-ExtraCare project. The provision of supported living accommodation for older adults in the UK is complex (we acknowledge systems are different in the US, in other European countries, and around the world). Issues include the separation of health and social care budgets, increased privatisation, limits to funding from Local Authorities, the Personal Independence Payment framework for people living with disability or long-term conditions, and the ever-present need to successfully demonstrate better health outcomes in order to receive government funding for the provision of care. Thus part of our remit, not described in this paper, involved the generation of health economics data, which demonstrated clear savings for the National Health Service in terms of reduced unplanned hospitalisation and general practice visits for ExtraCare residents (see: www.extracare.org.uk/research/findings). These findings were valuable and have since been used to change policy and to develop new research questions now being tackled as part of a Europe-wide study about the prevention and management of frailty among older adults (for some background about the frailty project, FOCUS, see: D’Avanzo et al., 2017; Gwyther et al., 2017, 2018 http://focus-aha.eu/home).

Finally, the first hurdle with MMR we considered at the start of this paper – that qualitative and quantitative data are like oil and water and are arguably incommensurate – simply disappears with MDR. By moving focus away from the types of data collected to (i) the research design, (ii) the possible research questions in a mixed design, (iii) the underlying paradigms, and (iv) the respective logics of inquiry, what results is a comprehensive programme of research with clearly articulated goals.

There is still work to be done to further test the Model of Disciplined Inquiry and to raise awareness of the logics of inquiry as fundamental to decision-making in study design. Nevertheless, there has been huge progress with increased openness in health psychology and behavioural medicine to expanding the evidence base. Researchers are being creative and exploring new ways of doing things to better our theoretical understanding but also to improve our research toolbox.
